# Economic Outcomes of Maintenance Gefitinib for Locally Advanced/Metastatic Non-Small-Cell Lung Cancer with Unknown EGFR Mutations: A Semi-Markov Model Analysis

**DOI:** 10.1371/journal.pone.0088881

**Published:** 2014-02-20

**Authors:** Xiaohui Zeng, Jianhe Li, Liubao Peng, Yunhua Wang, Chongqing Tan, Gannong Chen, Xiaomin Wan, Qiong Lu, Lidan Yi

**Affiliations:** 1 PET-CT Center, the Second Xiangya Hospital of Central South University, Changsha, Hunan, People’s Republic of China; 2 Department of Pharmacy, the Second Xiangya Hospital of Central South University, Changsha, Hunan, People’s Republic of China; 3 School of Pharmaceutical Sciences, Central South University, Changsha, Hunan, People’s Republic of China; 4 Department of Surgery, the Second Xiangya Hospital of Central South University, Changsha, Hunan, People’s Republic of China; Deutsches Krebsforschungszentrum, Germany

## Abstract

**Background:**

Maintenance gefitinib significantly prolonged progression-free survival (PFS) compared with placebo in patients from eastern Asian with locally advanced/metastatic non-small-cell lung cancer (NSCLC) after four chemotherapeutic cycles (21 days per cycle) of first-line platinum-based combination chemotherapy without disease progression. The objective of the current study was to evaluate the cost-effectiveness of maintenance gefitinib therapy after four chemotherapeutic cycle’s stand first-line platinum-based chemotherapy for patients with locally advanced or metastatic NSCLC with unknown EGFR mutations, from a Chinese health care system perspective.

**Methods and Findings:**

A semi-Markov model was designed to evaluate cost-effectiveness of the maintenance gefitinib treatment. Two-parametric Weibull and Log-logistic distribution were fitted to PFS and overall survival curves independently. One-way and probabilistic sensitivity analyses were conducted to assess the stability of the model designed. The model base-case analysis suggested that maintenance gefitinib would increase benefits in a 1, 3, 6 or 10-year time horizon, with incremental $184,829, $19,214, $19,328, and $21,308 per quality-adjusted life-year (QALY) gained, respectively. The most sensitive influential variable in the cost-effectiveness analysis was utility of PFS plus rash, followed by utility of PFS plus diarrhoea, utility of progressed disease, price of gefitinib, cost of follow-up treatment in progressed survival state, and utility of PFS on oral therapy. The price of gefitinib is the most significant parameter that could reduce the incremental cost per QALY. Probabilistic sensitivity analysis indicated that the cost-effective probability of maintenance gefitinib was zero under the willingness-to-pay (WTP) threshold of $16,349 (3×per-capita gross domestic product of China). The sensitivity analyses all suggested that the model was robust.

**Conclusions:**

Maintenance gefitinib following first-line platinum-based chemotherapy for patients with locally advanced/metastatic NSCLC with unknown EGFR mutations is not cost-effective. Decreasing the price of gefitinib may be a preferential choice for meeting widely treatment demands in China.

## Introduction

Lung cancer, the most commonly diagnosed form of cancer, is also the leading mortality cause of cancer in males [1]. Non-small-cell lung cancer (NSCLC) accounts for approximately 80% of all lung cancer cases, and the majority of patients with NSCLC have locally advanced/metastatic disease when they are diagnosed with carcinoma [2], [3]. Platinum-based combination therapies are recommended as first-line chemotherapy for unselected patients with locally advanced/metastatic NSCLC [4], [5]. However, the duration of them (4–6 chemotherapeutic cycles, 21 days per cycle) are limited by cumulative toxicities, and response rates (20%–35%) and median overall survival (7–12 months) are modest [6], [7]. On the basis of previous investigations, efforts to improve treatment outcome have focused on the specific goal of prolonging tumour response, progression-free survival (PFS) and overall survival (OS) with well tolerated maintenance treatment in patients who have attained tumor control during first-line treatment [8]–[12]. Because of these trials and other findings, both erlotinib and pemetrexed (for patients with histologies other than squamous cell carcinoma), have been approved by clinical guidelines as a category 2A recommendation for switch maintenance therapy and also been approved by FDA, in patients without disease progression after 4–6 chemotherapeutic cycles of first-line therapy [4], [13], [14].

In *The Lancet Oncology* recently, Li Zhang et al, based on a double-blind randomised phase 3 trial, reported that maintenance gefitinib significantly prolonged PFS compared with placebo in patients from 27 centres across China with locally/metastatic NSCLC, which indicates that gefitinib should be considered as a maintenance treatment choice in eastern Asian patients [15]. Several economic studies were conducted of maintenance therapy [16]–[23]. Two analyses concluded that maintenance erlotinib is cost-effective versus best supportive care for locally advanced/metastatic NSCLC [16], [17]. Except for the study by Greenhalgh et al [18], the 4 other studies of maintenance pemetrexed indicated that the new therapy was not cost-effective [19]–[22]. The evaluation from Zhu J et al, on the basis of the clinical trial, suggested that the maintenance gefitinib therapy was cost-effective for locally advanced/metastatic NSCLC patients with activating EGFR mutations [23]. However, it is unclear whether the new therapy is cost-effective in patients with unknown EGFR mutations after first-line platinum-based combination chemotherapy without disease progression.

The objective of the current study was to evaluate the long-term cost-effectiveness (10 year time horizon) of maintenance gefitinib therapy after four chemotherapeutic cycles of stand first-line platinum-based chemotherapy for locally advanced/metastatic NSCLC patients with unknown EGFR mutations, from a Chinese health care system perspective.

## Materials and Methods

A previously constructed semi-Markov model was used to compare the long-tern impact of maintenance gefitinib treatment versus placebo after 4 chemotherapeutic cycles of first-line platinum-based chemotherapy for patients with locally advanced/metastatic NSCLC [22], on the basis of the double-blind randomised phase III trial from China by Li Zhang et al [15]. The model along with two-parametric Weibull and Log-logistic distribution were used for calculating the direct medical costs, life-years gained (LYGs) and quality-adjusted life-years (QALYs) gained of the practice presented in the trial [15]. Due to the perspective of the Chinese health care system, only direct medical costs related to the practice were estimated, including maintenance gefitinib therapy, treatment of major adverse events, routine follow-up treatment for patients without progression, follow-up treatment for progressive disease and terminal-phase cost. Costs in this study were estimated in US dollars (USD), corresponding to the 2011 consumer price index and assuming an average exchange rate of 1 USD to 6.45 Chinese Yuan (RMB). Utilities for the model were derived from the literature. The future costs and outcomes were discounted at 3% annually in compliance with the request of China Guidelines for Pharmacoeconomic Evaluations (version 8) [24].

Effectiveness data were stemmed from the multicentre, double-blind randomised clinical trial [15], which is the only phase III trial compared maintenance gefitinib treatment in patients with locally advanced/metastatic NSCLC according to our literature search. In brief, 296 patients with histological or cytological NSCLC in stage IIIb or IV between September 28, 2008 and August 11, 2009, who were 18 years or older and had a WHO performance status of 0–2 and more than 12 weeks life expectancy after completion of four chemotherapeutic cycle’s first-line platinum-based chemotherapy without disease progression, were eligible for the maintenance gefitinib or placebo treatment (1∶1 randomization ratio). Eligible patients continued to take either gefitinib (250 mg per day) or placebo orally until disease progression, intolerable toxicity, withdrawal of consent, serious non-compliance with protocol, or dose delay or interruption >14 days. In this report, there were 40 and 39 patients were deemed know EGFR mutation status in gefitinib group and placebo group, respectively. Therefore there were 108 patients and 109 patients with unknown EGFR mutation received maintenance gefitinib and placebo treatment separately. The primary endpoint of the trial was progression-free survival, and the survival analysis revealed that median PFS for patients with unknown EGFR mutation was 6.0 months in gefitinib group and 2.7 months in placebo group (HR 0.40 [95% CI 0.29–0.54]; p<0.0001). Median OS was not significantly different between the two groups (HR 0.84 [95% CI 0.62–1.14]; p = 0.26; median OS 18.7 months *vs* 16.9 months). The incidence of adverse events in gefitinib group was more frequent than that in placebo group (80% vs. 53%). The cumulative probabilities of serious adverse events were 7% and 3% in the maintenance gefitinib and placebo groups, respectively.

The model outcomes were presented as costs, LYGs and QALYs, from the perspective of the Chinese health care system. Sensitivity analyses of input parameters with the high/low values and various distributions were conducted to assess the stability of the model at a value of recommended willingness-to-pay (WTP) threshold of $16,349 (3×per-capita gross domestic product, GDP), based on the cost-effectiveness guidelines of Word Health Organization (WHO) [25].

### Model Structure

**Figure 1:**
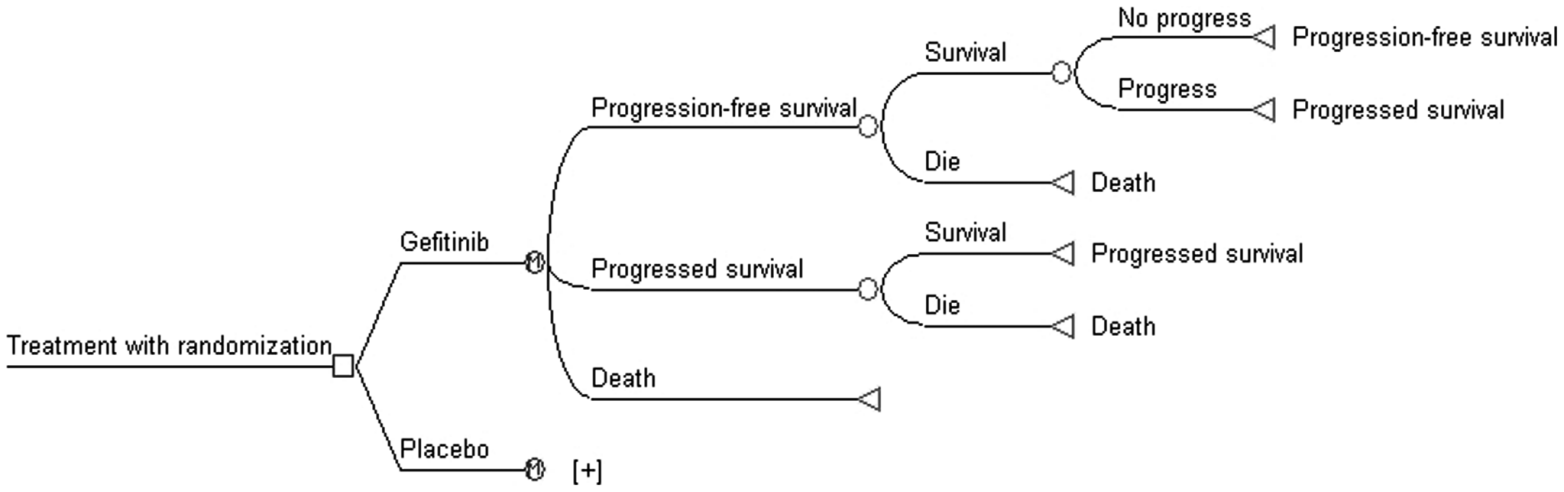
Markov model of locally advanced/metastatic non-small-cell lung cancer.

The simplified model structure was shown in Figure 1, which comprised 3 mutually exclusive health states: PFS (entry state); progressed survival (PS state), and death. Patients move from one state to another during each Markov cycle length of 3 weeks (short enough to detect all clinically relevant events) until time horizon termination of 10-year (>95% patients died). Two-parametric Weibull survival and Log-logistic distribution analyses using R for Statistical Computing version 2.15.2 (R Foundation, Wien, Austria) were fitted to the PFS and OS curves respectively, on the basis of survival data extracted from the published Kaplan-Meier curves [15], by using GetData Graph Digitizer software (version 2.24). Table 1 shows the Weibull and Log-logistic distribution parameters of model estimated. The estimated Weibull parameters are used to measure the time-dependency transition probabilities from PFS to PS state, according to the following formula:
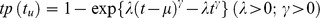
where the λ defines the scale of the distribution, the γ gives the shape, the u is the Markov cycle and t_u_ indicates that t is calculated as integer multiples of the cycle length of the model. The transition probabilities of death at current t due to the following formula:
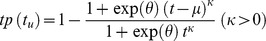
where the θ and κ are the theta and kappa from the estimated Log-logistic parameters, indications of the u and the t_u_ are the same as above.

**Table 1:** Weibull and Log-logistic parameters of model estimated for progression-free and overall survival curves, respectively.

Progression-free survival^a^	Scale, Mean (Range)	Shape, Mean (Range)	Adjusted R^2^	Correlation Coefficient
Placebo arm	0.10443 (0.04509/0.16377)	1.29221 (0.99662/1.58780)	0.9729	−0.995165
Gefitinib arm	0.10231 (0.06622/0.13840)	0.83852 (0.71474/0.96230)	0.9782	−0.998386
**Overall survival** b	**Theta, Mean (Range)**	**Kappa, Mean (Range)**	**Adjusted R^2^**	**Correlation Coefficient**
Placebo arm	−6.54311 (−7.16112/−5.92510)	2.09373 (1.89823/2.28923)	0.9855	−0.999986
Gefitinib arm	−5.04069 (−5.53622/−4.54516)	1.54139 (1.38359/1.69919)	0.9801	−0.999852

a R output for Weibull regression fitted to progression-free curves of locally advanced/metastatic non-small-cell lung cancer patients derived from the Phase III trial [15].

b R output for Log-logistic regression fitted to overall curves of locally advanced/metastatic non-small-cell lung cancer patients derived from the Phase III trial [15].

### Medical Costs and Utilities

Medical costs for each strategy (Table 2), from the perspective of Chinese health care system, were based on outlining current practice [15], which reflected the effectiveness of maintenance gefitinib treatment in Chinese patients with locally advanced/metastatic NSCLC. Direct medical costs related to the practice were estimated, including maintenance gefitinib therapy, treatment of major adverse events, routine follow-up treatment for patients without progression, follow-up treatment in PS state and terminal-phase cost. Prices of gefitinib, follow-up treatment cost in PS state and terminal-phase cost were obtained from our previous study, in which we have calculated healthcare costs associated with the time- and health status-related treatment resources that advanced NSCLC may anticipate based on health expenditure data for 253 cases of advanced NSCLC registered at the Second Xiangya Hospital of Central South University in China between 2006 and 2010 [26]. The aggregate annual medical costs for patients in either PFS or PS state and monthly healthcare costs accumulated during the terminal 3 months, were estimated and evaluated using 95% confidence intervals through bootstrapping with the R software (version 2.14.0; R Foundation, Vienna, Austria) [26]. According to Gefitinib Patients Assistance Program of the pharmaceutical producer in China, NSCLC patients receive donations of gefitinib after six months treatment [23]. Therefore six months was applied to calculate the total cost of the maintenance drug. Routine follow-up treatment cost for patients without progression, including computed tomography scan, physician visit, and other examinations and drugs, was derived from the literature by Wu B et al [27]. Based on expert opinion, only diarrhoea and other grade 3/4 adverse events were considered to estimate the costs of treatment-associated toxicity. Therefore the unit costs of diarrhoea treated and liver protected were multiplied by published rates of corresponding events to populate the model analysis (we assumed patients with grade 3/4 alanine aminotransferase, aspartate aminotransferase or aminotransferases increased should receive treatment of liver protected). The unit costs of diarrhoea and liver protected were estimated according to local charges in China.

**Table 2:** Base cases, ranges and distributional assumptions of parameters.

Variables	Base case	Range	Distribution
Costs ($)			
Treatment costs			
Gefitinib per 250 mg [26]	81.0	64.8/81.0^a^	Fixed in PSA
Routine follow-up of patients per unit [27]	51.5	45.0/58.4	Lognormal
Follow-up treatment in PS state each year [26]	14,519	12,011/16,871	Lognormal
Terminal phase cost in last month [26]	3754	3274/4238	Lognormal
Adverse events			
Liver protected per unit^b^	57.78	32.07/96.22	Lognormal
Diarrhoea per unit^b^	1.48	0.89/2.08	Lognormal
Utility values			
Progression-free survival on oral therapy [28]	0.67	0.27/0.80	Beta
Progression-free survival plus rash [28]	0.62	0.25/0.74	Beta
Progression-free survival plus diarrhoea [28]	0.61	0.24/0.73	Beta
Progressed disease survival [28]	0.47	0.19/0.56	Beta
Discount rate (%) [24]	3	0/8	Fixed in PSA
Risk for adverse events			
Rash in gefitinib arm [15]	0.50	0.35/0.65^c^	Beta
Rash in placebo arm [15]	0.095	0.067/0.124^c^	Beta
Diarrhoea in gefitinib arm [15]	0.25	0.18/0.32^c^	Beta
Diarrhoea in placebo arm [15]	0.088	0.062/0.114^c^	Beta
ALT increased in gefitinib arm (grade 3,4) [15]	0.020	0.014/0.026^c^	Beta
AST increased in gefitinib arm (grade 3,4) [15]	0.007	0.0049/0.0091^c^	Beta
ATR increased in gefitinib arm (grade 3,4) [15]	0.014	0.0098/0.0182^c^	Beta

PSA = Probabilistic sensitivity analysis; ALT = Alanine aminotransferase;

AST = Aspartate aminotransferase; ATR = Aminotransferases.

a Price of gefitinib was reduced 20% and was fixed in probabilistic sensitivity analysis because it is a brand name drug.

b Estimated according to local charges in China.

c Varied by ±30%.

Health state utility values of the PFS and PS states presented in Table 2 were derived from the published literature by Nafees et al, who assessed quality of life using the visual analogue scale and standard gamble interview in 100 participants, on the basis of the health state descriptions which were developed after rounds of in-depth interviews with oncologists, oncology specialist nurses and psychometric experts [28]. According to the literature, diarrhoea and rash reported in the trial, were significantly associated with the utility values of PFS [15], [28]. Therefore we calculated the utility value in PFS based on the published proportion of the adverse events (diarrhoea and rash) [15] and utility values of PFS on oral therapy (0.67), PFS plus rash (0.62), and PFS plus diarrhoea (0.61) [28]. The utility of PS state was 0.47 (range, 0.19–0.56) and was used in both arms.

### Sensitivity Analysis

Each key parameter was fitted high/low values and specific pattern of distribution in our model (Table 2) to reflect substantial uncertainty of the input parameters using one-way and probabilistic sensitivity analyses (PSA). Many ranges were derived from published reports [26]–[28]; price of gefitinib was reduced 20% and fixed in PSA because it is a brand name drug; costs of adverse events were estimated by local charges in China; probabilities of adverse events were varied by ±30%. Lognormal distributions were chosen for all input costs except gefitinib (fixed in PSA); beta distributions were chosen for utility values and probabilities of adverse events; bivariate normal distributions were adopted for the Weibull and Log-logistic parameters; discount rate with high/low values was fixed in PSA, in compliance with the request of China Guidelines for Pharmacoeconomic Evaluations (Version 8) [24]. The WTP threshold of 3×per-capita GDP ($16,349/QALY) was used. A tornado diagram and an incremental cost-effectiveness scatter plot were developed to depict the results of one-way sensitivity analyses (OSA) and PSA.

## Results

### Base Case Model Analysis

**Figure 2:**
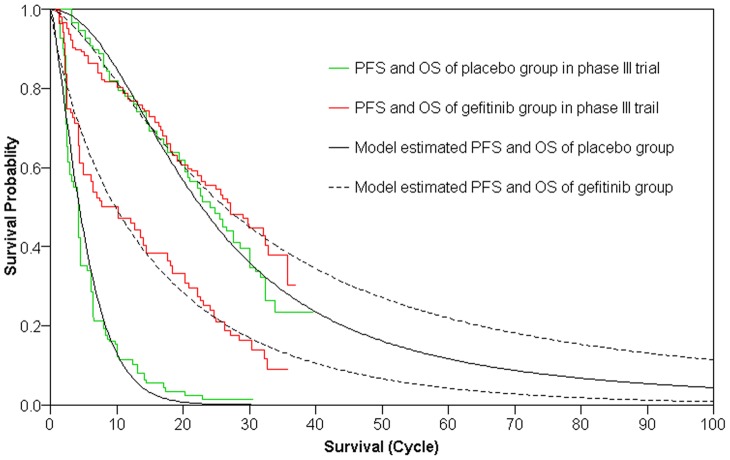
Survival curves in patients with maintenance gefitinib group or placebo group after first-line platinum-based combination chemotherapy of four cycles in locally advanced/metastatic non-small-cell lung cancer. The original curves from the clinical trial are shown, together with the Weibull and Log-logistic model estimated for progression-free survival and overall survival separately.

The Log-logistic and two parameters Weibull model matched the survival curves of the clinical trial by Zhang L et al [15] satisfactorily (Figure 2). The validity of the simulated survival curves tail beyond the observed time horizon of clinical trial was conducted by comparing the 5 years overall survival rate calculated from the current distribution models for placebo arm (5.7%) to the data from the Surveillance, Epidemiology and End Results (SEER) Program, which shows that 5-year survival rate of distant lung cancer patients is 3.9% [29], and the site of http://lungcancer.about.com/, which shows that 5-year survival rate of metastatic NSCLC is sadly less than 10%. Base case model analyses in different time horizon are displayed in Table 3, which suggested that maintenance gefitinib therapy after four chemotherapeutic cycles of stand first-line platinum-based chemotherapy for patients with locally advanced/metastatic NSCLC patients would increase the effectiveness in a 1-, 3-, 6-, or 10-year time horizon, with incremental QALYs gained of 0.0233, 0.1462, 0.2699 and 0.3496. Incremental costs per QALY for the new therapy compared with placebo were $184,828, $19,214, $19,328 and $21,308, respectively, at 1, 3, 6, and 10 years.

**Table 3:** Base-case model analyses of life-years gained (LYGs), quality-adjusted life-years (QALYs), costs and incremental cost per LYG/QALY of maintenance gefitinib therapy arm and placebo arm after four cycles of stand first-line platinum-based chemotherapy for patients with locally advanced/metastatic NSCLC, on the basis of 1000 simulation cases.

Arm	LYGs Gained	QALYs Gained	Cost ($)	Incremental cost ($)	
				Per LYG	Per QALY
1-year					
Placebo arm	0.865	0.465	9,082	–	–
Gefitinib arm	0.845	0.488	13,396	Dominated	184,829
3-year					
Placebo arm	1.516	0.772	18,866	–	–
Gefitinib arm	1.658	0.918	21,675	19,788	19,214
6-year					
Placebo arm	1.735	0.875	22,129	–	–
Gefitinib arm	2.112	1.144	27,345	13,816	19,328
10-year					
Placebo arm	1.814	0.912	23,302	–	–
Gefitinib arm	2.357	1.262	30,751	13,734	21,308

### One-way Sensitivity Analysis

**Figure 3:**
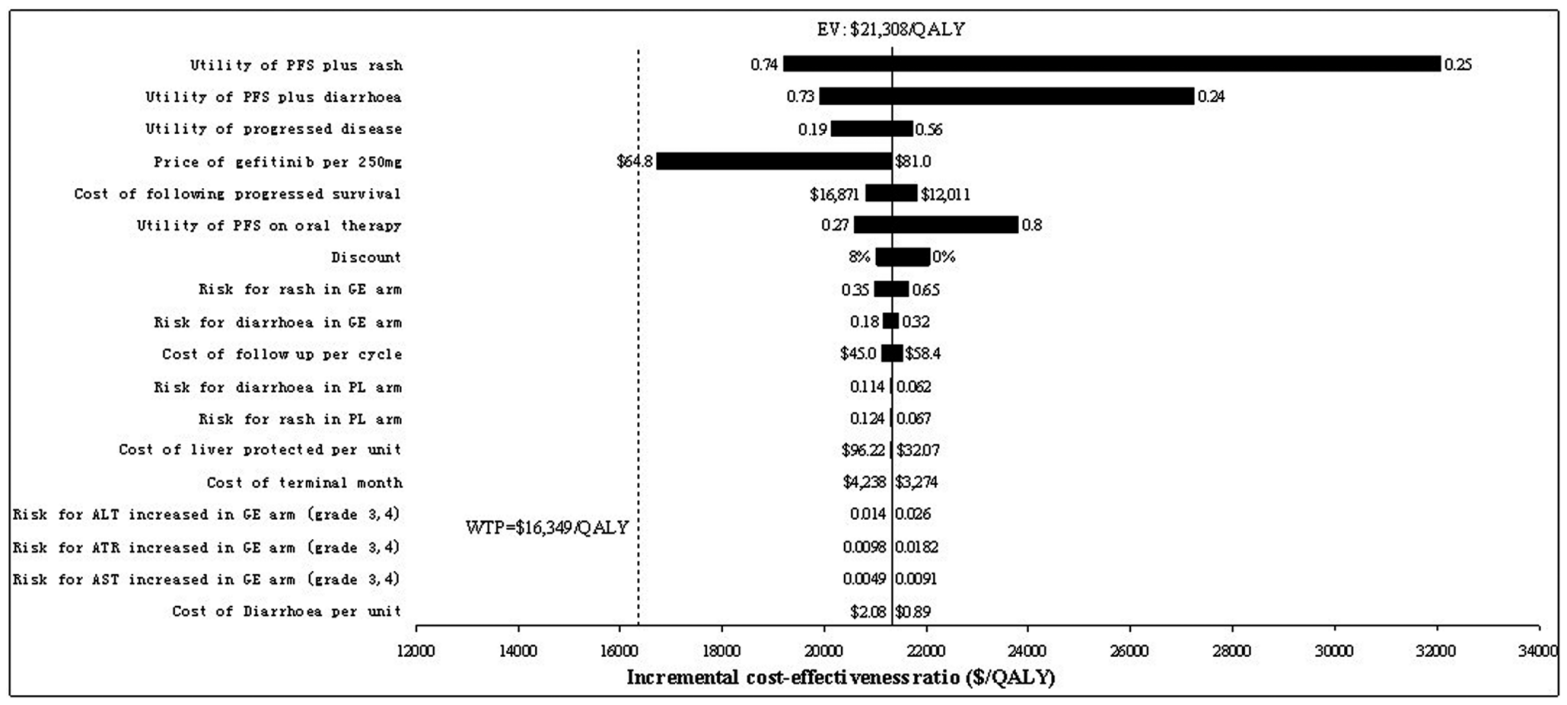
Tornado diagram for one-way sensitivity analysis revealing variables’ influence on the incremental cost-effectiveness ratio. PFS = progression-free survival; GE = gefitinib; PL = placebo; QALY = quality-adjusted life-years.

The results of one-way sensitivity analyses of key populated variables (displayed in Table 2) were depicted in a tornado diagram (Figure 3) to show the influence with regard to the incremental cost-effectiveness ratio (ICER), which means incremental cost per QALY gained in current study. The utility of PFS plus rash impacted utmost on the ICER. The other sensitive variables included utility of PFS plus diarrhoea, utility of progressed disease, price of gefitinib, cost of follow-up treatment in PS state, and utility of PFS on oral therapy. All of these variables did not led to an ICER entrancing the WTP threshold of $16,349 per QALY (3×per-capita GDP of China). None of the other parameters significantly altered the ICER.

### Probabilistic Sensitivity Analysis

**Figure 4:**
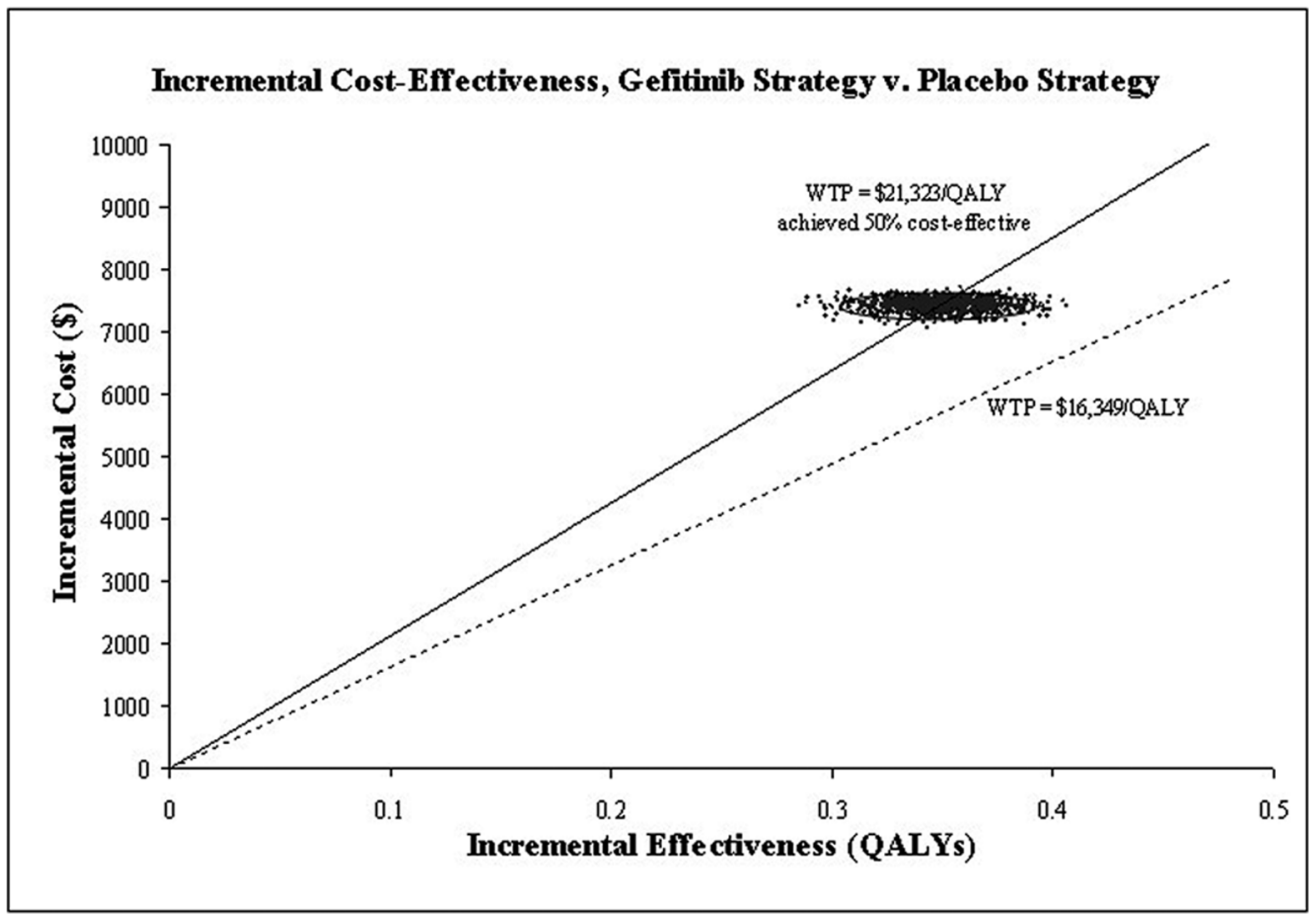
Probabilistic sensitivity analysis of 1000 cases study comparing maintenance gefitinib strategy and placebo strategy. WTP = willingness to pay; QALY = quality-adjusted life-years.

**Figure 5:**
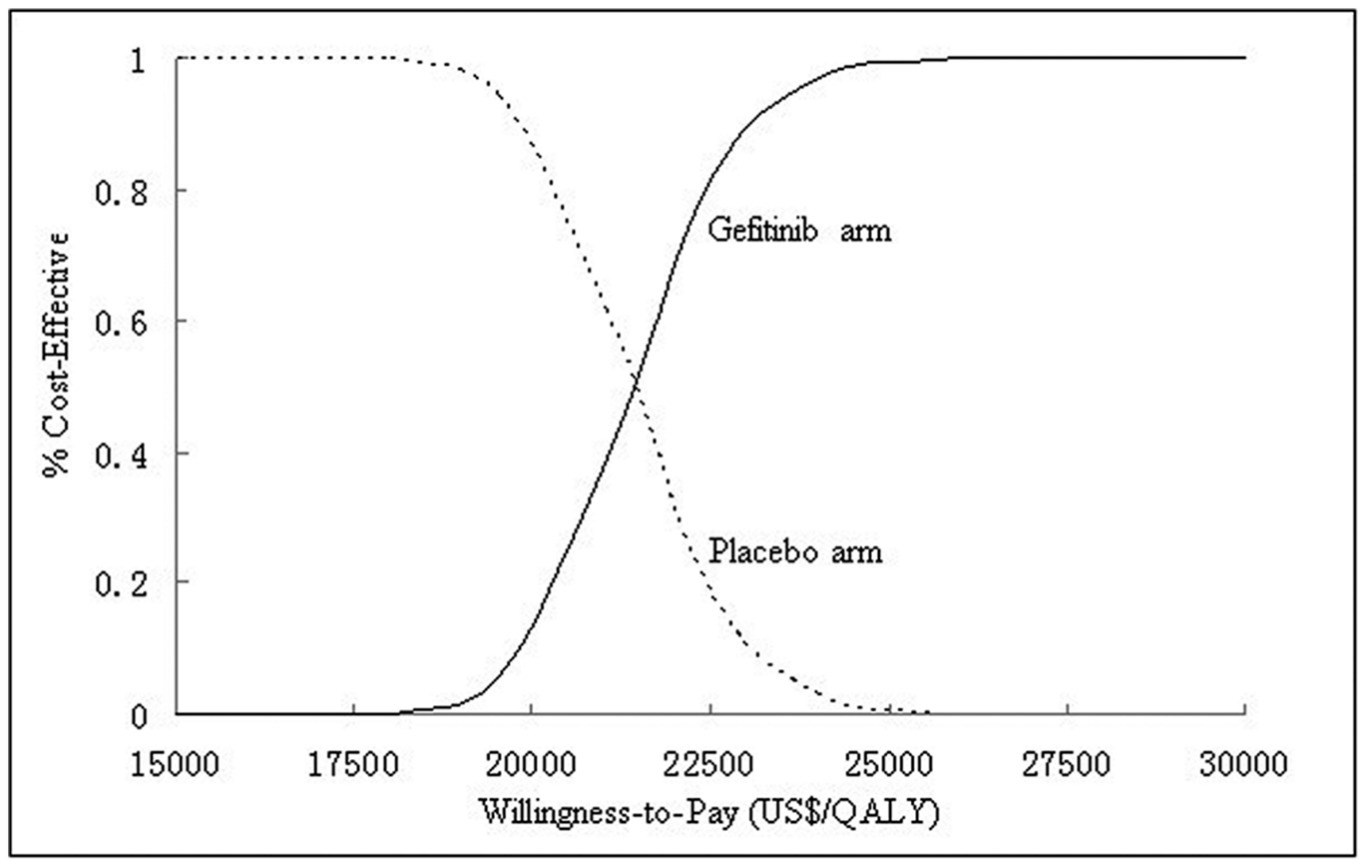
Acceptability curves of maintenance gefitinib arm and placebo arm. QALY = quality-adjusted life-years.

Incremental cost-effectiveness scatter plot of 1000 simulations (Figure 4), showed a zero probability meeting the WTP threshold of $16,349/QALY. If the WTP was >$21,323 (per-capita GDP: $7,108), more 50% of locally advanced/metastatic NSCLC, with maintenance gefitinib therapy after four cycles of first-line platinum-based combination chemotherapy without disease progression, could achieve cost-effectiveness. Acceptability curves (Figure 5) suggested that the cost-effectiveness likelihood of maintenance gefitinib therapy increased with increasing thresholds of WTP, and about $17,700 to $26,300 was the sensitivity range. At WTPs >$26,300, almost all cases could achieve cost-effectiveness.

## Discussion

Maintenance gefitinib therapy has been proven to prolong PFS significantly than placebo for patients with locally advanced/metastatic NSCLC after 4 chemotherapeutic cycles of first-line platinum-based combination chemotherapy without disease progression, based on a Chinese phase III trial across 27 centres [15]. However, its economic impact is necessary to be considered before it is widely used for the appropriate patients, especially for China, where the population is >1.3 billion and the health care resources are in serious shortage [30].

Resource allocation decisions in health care are rife based on results from economic assessments. However from Clinical trials, it is of difficulty to collect enough financial data for economic evaluation [27]. Thus, mathematical models that can estimate long-term cost-effectiveness of alternative strategies, is a helpful technique to support economic analyses of health care resource ulitization [26], [27]. In current study, a semi-Markov model along with two-parametric Weibull and Log-logistic distribution were used for measuring the time-dependency transition probabilities and calculating the direct medical costs, LYGs and QALYs gained of the practice presented in the trial [15].

A cost-effectiveness evaluation was performed to analysis the economic impact of maintenance gefitinib therapy for patients with locally advanced/metastatic NSCLC with unknown EGFR mutations. Base case analyses of 1-, 3-, 6-, and 10-year time horizon showed an unfavorable ICER of $184,829, $19,214, $19,328, and $21,308 per QALY gained, respectively. OSA and PSA all revealed that the model we applied was robust to the results. Monte Carlo simulations of 1,000 cases suggested that all ICERs for maintenance gefitinib therapy were higher than the recommended WTP threshold (3×per-capita GDP) of cost-effectiveness guidelines from Word Health Organization (WHO). There are 31 province-level administrative units in Chinese mainland, the per-capita GDP of which differs significantly. In 2011, for example, it ranged from $2,495 in Guizhou province to $13,392 in Tianjin city [31]. According to the recommended threshold of WHO [25], the WTP threshold, of different province-level administrative units, extended from $7,485 (3×$2,495) to $40,176 (3×$13,392) per QALY gained, which exceeded the sensitivity range of the WTP (about $17,700 to $26,300) obtained from PSA of the current study. Obviously local government could take fully into account covering maintenance gefitinib treatment following first-line platinum-based chemotherapy for locally advanced/metastatic NSCLC with unknown EGFR mutations in accordance with local economic development level. Cost-effective probability for different economic level provinces, displayed in Table 4, could supply available information for local governments, when gefitinib is approved by local governments’ finance before it has access to the directory of drugs for national basic medical insurance in China.

**Table 4:** The cost-effective probabilities of gefitinib arm for 31 provinces of Chinese mainland.

Region	Per-capita GDP ($)	WTP (3×Per-capita GDP, $)	Cost-effective Probability
Mainland China	5,449.71	16,349	0
More affluent regions^a^	>8,767	>26,300	1.00
Guangdong	7,819	23,457	0.932
Liaoning	7,795	23,385	0.926
Fujian	7,344	22,032	0.717
Shandong	7,273	21,819	0.655
Less affluent regions^b^	<5,900	<17,700	0

a Consist of Tianjin, Shanghai, Beijing, Jiangsu, Zhejiang and Inner Mongolia.

b Consist of Jilin, Chongqing, Hubei, Hebei, Shanxi, Ningxia, Heilongjiang, Shangxi, Xinjiang, Hunan, Qinghai, Henan, Hainan, Jiangxi, Sichuan, Guangxi, Anhui, Tibet, Gansu, Yuannan and Guizhou.

A number of different survival models, such as Weibull, Exponential, Log-logistic, Gompertz, et al, can be used to perform extrapolation according to the observed trial data [32]. It is therefore very vital to choose the justifiable extrapolation approach, to ensure the associated results of economic analysis confident to decision makers. In the current study, after the deviance information criterion test (reported by Jackson et al [33] to alternative models introduced by Latimer [32], we chose Weibull and Log-logistic for PFS and OS respectively, instead of Weibull for extrapolating both PFS and OS curves like the previous study undertaken by Zhu J et al [23]. In addition, a hazard ration (HR) of PFS was applied to derive the PFS curve for the gefitinib strategy in the previous study [23]. Latimer, however in the resent published paper, pointed out that the HR used may cause bias because of the requirement of the assumptions–that is, the HR was from a related model and was constant over time [34]. Obviously the bias should be considered, especially if the HR impacts the results markedly. Unfortunately, the HR of PFS was one of the two most influential parameters on the basis of one-way sensitivity analyses performed by Zhu J et al [23]. In view of the above cases, independent parametric models were fitted to both control and experimental groups in our study.

Utility of PFS played a great role in the results not only in the resent study [23] but also in the current study. Nafees et al [28] reviewed that all toxicities (diarrhoea, rash, nausea and vomiting, neutropenia, fatigue, and hair loss) were related to pulling utility down significantly. Of the toxicities, rash and diarrhoea were associated with maintenance gefitinib strategy as reported the clinical trial [15]. For higher accuracy, we weighted the utility of PFS according to the risks of the rash and diarrhoea, which were displayed in Table 2.

In particularly, one point revealed by one-way sensitivity analysis (Figure 3), should be highlighted that the price of gefitinib would be the most significant parameter that could reduce the ICER. With the gefitinib price reduction of 20% discount, the ICER decreased to $16,731 per QALY gained, which is very close to the WTP threshold of $16,349 per QALY. Therefore if the price of gefitinib decreases >20%, maintenance gefitinib therapy after the standard chemotherapy in patients with locally advanced/metastatic NSCLC may be a cost-effectiveness strategy.

There are some limitations in the present study. First, using Weibull and Log-logistic distribution to extrapolate the survival curves beyond the time scope of the trial was an unavoidable limitation of this process. There is not enough survival data, provided by the short follow-ups of the clinical trial, to compare the long-term outcomes estimated by the model. Our results should be updated when long-term survival data are available. Another important limitation is that the utility weight parameters originated from the published literature that may not reflect Chinese patients’ trait. It is an inevitable limitation of the current analysis because utilities data are not yet available for China. Fortunately, opinions from Chinese oncologists suggested that, quality of life of locally advanced or metastatic NSCLC patients in China should not be of significant difference from abroad patients. Finally, because there is no head-to-head clinical trial comparing maintenance gefitinib with other maintenance drugs (eg, erlotinib) after the standard chemotherapy of four chemotherapeutic cycles, we have not conducted a cost-effectiveness analysis of gefitinib in comparison with other maintenance therapies.

Although the current estimates were derived from just one study, which is also the only phase III trial compared maintenance gefitinib treatment in patients with locally advanced/metastatic NSCLC according to our literature search, we believe that the analysis of our study, based on a current Chinese phase III trial and the justifiable extrapolation approach, can provide important reference information for decision makers in China. First of all, the clinical study itself is a multicentre, double-blind, randomized controlled-trial (RCT), which represents the best evidence available and is deemed to be the most accepted scientific method of determining the benefit of a drug or a therapeutic procedure. Second, the analysis method applied in our study was reliable and widely used in economic evaluations, especially in the field of medical and health care. In addition, the Log-logistic and two parameters Weibull model matched the survival curves of the clinical trial satisfactorily (Figure 2), which shows that the model we constructed can mirror the effectiveness data of the trial commendably. And then, direct medical costs related to each strategy were estimated, including maintenance gefitinib therapy, treatment of major adverse events, routine follow-up treatment for patients without progression, follow-up treatment in PS state and terminal-phase cost. Although the costs originated from our previous study [26], the published literature [27] or estimates according to local charges based on expert opinion, all of them stemmed from a Chinese health care system perspective, as well as in view of patients with advanced NSCLC, which echoed the purpose of the current study. Last but not least, to reflect substantial uncertainty of the input parameters, the sensitivity analyses (including OSA and PSA) were conducted for each key parameter, and all sensitivity analyses revealed that the model we applied was robust to the results.

In conclusion, according to the recommended WTP threshold (3×per-capita GDP) of cost-effectiveness guidelines from WHO, maintenance gefitinib therapy after the standard chemotherapy of four chemotherapeutic cycles in locally advanced/metastatic NSCLC patients with unknown EGFR mutations is likely to be not cost-effective for Chinese mainland, from the Chinese health care system perspective. Local governments, with different economic level however, could take fully into account covering maintenance gefitinib treatment. Because for rich regions (the per-capita GDP> $8,767), the new strategy seems to be a reasonable option, and if the per-capita GDP ranges from $5,900 to $8,767, the maintenance therapy may be favourable in terms of the different cost-effective probabilities. Decreasing the price of gefitinib, the most significant parameter that could reduce the ICER, should be considered to as a preferential factor for meeting widely treatment demands in China.
